# Fabrication of Anti-Fatigue Double-Wrapped Yarns with Excellent Mechanical Properties for Generating Compression Fabrics

**DOI:** 10.3390/polym16172476

**Published:** 2024-08-30

**Authors:** Qian Zhang, Jiaqi Chen, Ziqin He, Wenfu Liu, Andreii S. Kritchenkov, Lu Wang, Wanjun Liu, Jing Gao

**Affiliations:** 1 Key Laboratory of Textile Science & Technology, Ministry of Education, College of Textiles, Donghua University, Shanghai 201620, China; 2 Engineering Research Center of Technical Textiles, Ministry of Education, College of Textiles, Donghua University, Shanghai 201620, China; 3 Shanghai Engineering Research Center of Nano-Biomaterials and Regenerative Medicine, Donghua University, Shanghai 201620, China; 4 College of Energy Engineering, Huanghuai University, Zhumadian 463000, China; liuwenfu@huanghuai.edu.cn; 5 Faculty of Science, Peoples’ Friendship University of Russia (RUDN University), 117198 Moscow, Russia; 6 Institute of Technical Acoustics NAS of Belarus, 210009 Vitebsk, Belarus

**Keywords:** double-wrapped yarns, anti-fatigue properties, manufacturing parameters, tensile property, weft-knitted fabrics, compression garments

## Abstract

Elastic yarns are the key component of high-performance compression garments. However, it remains a challenge to fabricate anti-fatigue yarns with high mechanical force and long elongation for generating compression garments with prolonged wear. In this paper, we report the development of anti-fatigue double-wrapped yarns with excellent mechanical properties by wrapping high-denier Spandex with nylon filaments in opposite twists. In particular, high-denier (560 D) Spandex as the core was untwisted, which can maximally reduce the interaction between the core and wrapping filaments, enabling high elongation of double-wrapped yarns. In addition, we chose 70 D nylon filaments with a tensile force of 3.87 ± 0.09 N as the wrapping materials to provide sufficient force for double-wrapped yarns. Notably, opposite twists were induced for the inner and outer wrapping filaments to achieve a balanced stable yarn structure. By systematically optimizing manufacturing parameters, including inner wrapping density, outer wrapping density, take-up ratio, and drafting ratio, we obtained double-wrapped yarn with excellent tensile stress (32.59 ± 0.82 MPa) and tensile strain (357.28% ± 9.10%). Notably, the stress decay rate of optimized yarns was only 12.0% ± 2.2%. In addition, the optimized yarn was used as the weft-lining yarn for generating weft-lined fabrics. The elastic recovery rate of the obtained fabric was decreased by only 2.6% after five cyclic stretches, much lower than the control fabric. Our design of anti-fatigue double-wrapped yarns could be widely used for fabricating high-performance compression garments.

## 1. Introduction

High-elastic yarns are a key raw material for the production of compression garments that can provide stability, compression, and support to the human body [1]. Such compression garments have been widely used in the medical, sports, and aerospace fields in the form of compression stockings and sleeves, orthopedic supports, and functional sportswear [2,3,4]. In particular, compression stockings and sleeves are worn to apply pressure to the skin and deep soft tissue, thereby narrowing the width of veins and promoting the reabsorption of lymphatic fluid [5,6]. Therefore, compression stockings and sleeves can be used for treating chronic venous disorders including varicose veins, chronic edema, and deep vein thrombosis [7], avoiding require expensive therapy, and have no side effects compared to surgical and pharmacological treatments [8]. In addition, compression garments can be used for treating burn wounds, providing an ischemic and hypoxic environment with the help of pressure and inhibiting the formation of hypertrophic scarring by impairing fibroblast growth and transformation [9,10]. Moreover, compression garments can reduce soft tissue vibration to improve joint stability, alter proprioceptive feedback and neuromuscular control, and facilitate metabolite clearance [11,12], enabling better athletic performance [13].

As the key component of high-performance compression garments, elastic yarns with good extensibility and elastic recovery allow garments to become effective stretching elements acting on the curved body, exerting the desired pressure on the surface of the body zone needing this [14]. However, Spandex elastic yarns usually have an elongation of 400–800%, but their elastic modulus is only 0.2–1.1 cN/tex [15], making it difficult to control tension during knitting, frequently resulting in fabric horizontal strips, uneven elasticity, and yarn breakage [16]. Therefore, Spandex is usually combined with other types of fibers to make composite yarns for better weaving performance, which can greatly increase the elastic modulus and yarn processability [17]. Presently, the most commonly used elastic composite yarns include core-spun yarns and wrapped yarns [18]. For example, nylon–Spandex [19] and cotton–Spandex [20] core-spun yarns usually have a tensile force of 5.88 N and 10.05 N, respectively, which can provide sufficiently high pressure. However, their elongation is in the range of 10–30%, which is too low to accommodate the wide range of body sizes and would restrict body movement and apparel moldability [21]. In addition, the elongation will be even less (6–12%) for double-core-spun yarns [22,23] and triple-core-spun yarns [24]. The low elongation is usually attributed to the strong cohesiveness between the core filament and sheath fibers for core-spun yarns due to the existence of twists in both components. It should be noted that more than 200% elongation is needed for regular compression garments.

In contrast, wrapped yarns have an untwisted core with a distinct core-to-sheath relationship, so the wrapped and core material cannot restrict each other’s elongation. Wrapping materials are usually selected from high-force filaments such as polyester [25] and nylon [26]. For example, the tensile elongation of nylon–Spandex-wrapped yarns can be up to 605%, but the tensile force is only 2.39 N [26], which is difficult to use for high-compression garments to generate a required pressure above 2666.44 Pa [14]. As reported, the tensile force of elastic inlay yarns should be above 4 N so that compression garments produce the desired pressure at about 30% stretch [27]. In addition, compression garments need to be used more than 50 times and are susceptible to pressure mismatch and relaxation fatigue [28]. For example, interlock-knitted fabrics with textured polyester yarns showed a pressure reduction of 22.5% after stretching for 48 h [29]. Even worse, the elastic recovery of single-knitted fabrics prepared from core-spun yarn was only 60.06% at 50% stretch [30]. The significant reduction in yarn strength and elasticity results from yarn fatigue [31]. Therefore, it is imperative to innovate on structure design of wrapped yarns to overcome yarn fatigue. To this end, it remains a challenge to fabricate anti-fatigue yarns with high mechanical force and long elongation for generating compression garments with prolonged wear.

In this study, we report the development of anti-fatigue double-wrapped yarns with excellent mechanical properties by wrapping high-denier Spandex with nylon filaments in opposite twists. We hypothesized that the interfaces formed by the core filament, inner wrapping filaments, and outer wrapping filaments would synergistically affect yarn mechanical properties. Specifically, we believed that there would be an appropriate wrapping angle (the angle between the wrapping filament and the axis of the yarn) and wrapping density to enable double-wrapped yarns with high mechanical force and large elongation simultaneously. By systematically optimizing the inner wrapping density, outer wrapping density, take-up ratio, and drafting ratio, we were able to precisely control the yarn structure and obtain double-wrapped yarns with outstanding tensile properties. We believe that these anti-fatigue double-wrapped yarns have the potential for widespread application in the production of compression garments.

## 2. Materials and Methods

### 2.1. Materials

Spandex filament was purchased from Haining Kaiwei Textile Co. (Jiaxing, China). Nylon filament was purchased from Huizhou Xinrongchang Yarn Co. (Huizhou, China). Their characteristics are presented in Table 1.

### 2.2. Fabrication of Double-Wrapped Yarns

**Figure 1:**
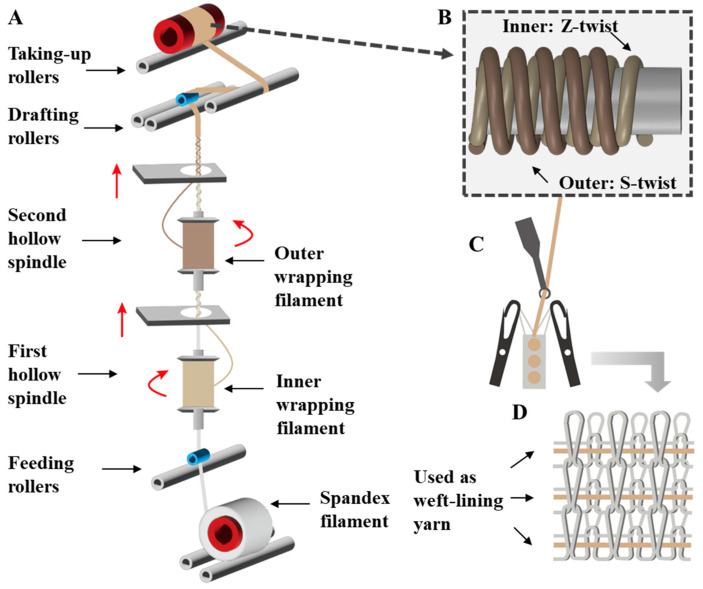
Design of double-wrapped yarns and weft-lined knitted fabric. (**A**) Schematic diagram showing the spinning of double-wrapped yarns, the red arrows indicates the moving direction of wrapped filament; (**B**) structure of the double-wrapped yarn; (**C**) schematic diagram showing the weft-lined fabric knitting process; (**D**) schematic diagram showing the structure of the weft-lined knitted fabric.

An HKV141A(II) yarn-wrapping machine (Zhejiang Jinggong Co., Ltd., Shaoxing, China) was used to fabricate double-wrapped yarns (Figure 1A). The wrapping process was divided into three parts: yarn core feeding, stretch wrapping, and loose winding. During the fabrication process, the Spandex filament was threaded into the cores of the two hollow spindles through the feeding rollers. The Z-twist and S-twist of nylon filaments were generated by the first and second hollow spindles, forming the inner and outer wrapping materials. Finally, the yarn was wound by take-up rollers. It should be noted that the wrapping density was controlled by the rotating speed of hollow spindles and the speed of drafting rollers, the drafting ratio was controlled by the speed ratio of drafting rollers and feeding rollers, and the take-up ratio was controlled by the speed ratio of take-up rollers and drafting rollers. It was wrapped twice at a certain drafting ratio and produced from the top drafting rollers. Each manufacturing parameter is defined below.

$$\mathrm{Inner}\;\mathrm{wrapping}\;\mathrm{density}\;\left(T/m\right)=\frac{\mathrm{Inner}\;\mathrm{nylon}\;\mathrm{spindle}\;\mathrm{speed}\;\left(\mathrm{rpm}\right)}{\mathrm{Drafting}\;\mathrm{rollers}\;\left(m/\min\right)}\;\mathrm{Outer}\;\mathrm{wrapping}\;\mathrm{density}\;\left(T/m\right)=\frac{\mathrm{Outer}\;\mathrm{nylon}\;\mathrm{spindle}\;\mathrm{speed}\;\left(\mathrm{rpm}\right)}{\mathrm{Drafting}\;\mathrm{rollers}\;\left(m/\min\right)}\;\mathrm{Taking}-\mathrm{up}\;\mathrm{ratio}\;=\frac{\mathrm{Taking}-\mathrm{up}\;\mathrm{rollers}\;\left(m/\min\right)}{\mathrm{Drafting}\;\mathrm{rollers}\;\left(m/\min\right)}\;\mathrm{Drafting}\;\mathrm{ratio}\;=\frac{\mathrm{Drafting}\;\mathrm{rollers}\;\left(m/\min\right)}{\mathrm{Feeding}\;\mathrm{rollers}\;\left(m/\min\right)}\;$$

### 2.3. Fabrication of Weft-Lined Knitted Fabrics

Both optimized and control yarns were used as the weft-lining yarn for generating weft-lined fabrics using a computerized flat knitting machine (Stoll ADF 530-32W), which was purchased from KARL MAYER STOLL Textilmaschinenfabrik GmbH (Reutlinge, Germany). Untwisted 140 D nylon filaments (48 f, Huizhou, China) and double-wrapped yarn (Huizhou, China) were used as the face yarn and plating yarn. The double-wrapped yarn was made from 40 D Spandex as the core and 70 D nylon filaments (48 f) as the double-wrapping materials to ensure that the ground fabric was elastic. A row of flat stitches was also added to the back of the fabric to improve the tightness. During the knitting process, the feed tension of the wrapped yarns was set to 0.08 N.

### 2.4. Characterizations

To acquire accurate testing results, all the wrapped yarn and fabric samples were conditioned for 24 h at 20 °C ± 2 °C and 65% ± 4% relative humidity following the standard of ISO 139:2005 (Textiles-Standard atmospheres for conditioning and testing) [32]. The yarn and fabric structures were examined with a DXS-10ACKT scanning electron microscope (Tianjin Electron Optics, Tianjin, China). For the tensile test, Spandex filaments and double-wrapped yarns were tested according to the standard of FZ/T 50006-2013 (Testing method for tenacity of Spandex filament yarns) [33], while nylon filaments were tested according to the standard of GB/T 3916-2013 (Textiles—Yarns from packages: Determination of single-end breaking force and elongation at break using constant rate of extension (CRE) tester) [34], both using the YG061F electronic single yarn force tester (Laizhou Electron Instrument, Laizhou, China). The breaking stress and breaking strain were quantified according to the stress–strain curves obtained. Each yarn sample was tested under a gauge length of 0.1 m and a prestressing value of 0.1 N at a speed of 0.0083 m/s, and each group was tested 10 times.

The elastic recovery rate and stress decay rate of double-wrapped yarns were measured using the YG061FQ electronic tensile tester (Laizhou Electron Instrument, Laizhou, China) through a constant elongation test following the FZ/T 50007-2012 standard (Testing method for the elasticity of spandex filament yarns) [35]. Each yarn sample was stretched to 0.15 m under a gauge length of 0.05 m and a prestressing value of 0.3 N at a speed of 0.0083 m/s. During the test, each sample was paused at 300% elongation and starting position for 10 s, and each group was tested 10 times.

The force and elastic recovery rate of knitted fabrics were tested using a YG(B) 026G-500 multi-functional force tester (Wenzhou Darong Textile Instrument Co., Ltd., Wenzhou, China) following the FZ/T 70006-2004 standard (Stretch and recovery testing method for knits) [36]. The sample was trimmed to a size of 0.2 m × 0.05 m and tested under a gauge length of 0.1 m and a prestressing value of 0.01 N at a stretching speed of 0.0017 m/s and recovery speed of 0.00017 m/s. During the test, each sample was paused at the designed elongation (i.e., 10%, 30%, 50%, 100%) and starting position for 30 s. This process was repeated 5 times, and each group was tested 10 times.

### 2.5. Queueing Scoring Rule

The score range for each mechanical property was determined by the number of parameter levels. For example, the score range of inner wrapping density and take-up ratio was 1–3 points, and the score range of outer wrapping density and drafting ratio was 1–5 points. Each level was assigned a score according to the value of mechanical properties, such as tensile force and breaking elongation. Finally, the sum of the scores for each level were calculated and the level with the highest score taken as the optimal parameter.

### 2.6. Statistical Evaluation

The statistical evaluation of the experimental results was performed using SPSS 26.0 statistical software. One-way analysis of variance (ANOVA) was performed to investigate the differences between the levels of parameters. Multiple-comparison least significant difference (LSD) tests were used to compare the means of performance based on the significance level. The difference in performance between the optimized yarns and the commercial yarns was analyzed using a paired t-test. All the analyses were performed at a significance level of α = 0.05. The confidence interval was 95% and the tolerance limit ±1.96 × S [37].

## 3. Results and Discussion

### 3.1. Design of Double-Wrapped Yarns and Weft-Lined Knitted Fabric

We designed double-wrapped yarns with an untwisted core of Spandex and double-wrapped nylon filaments in opposite twists for generating compression fabrics (Figure 1). In particular, high-denier (560 D) Spandex as the core was untwisted, which can maximally reduce the interaction between the core and wrapping filaments, enabling high elongation of the double-wrapped yarns. In addition, we chose 70 D nylon filaments with a tensile force of 3.87 ± 0.09 N as the wrapping materials to provide sufficient force for the double-wrapped yarns. Notably, opposite twists were induced for the inner and outer wrapping filaments to achieve a balanced stable yarn structure.

During the fabrication, the Spandex filament was placed at the bottom of the machine and sequentially passed through the feeding rollers, the holes of the first and the second hollow spindles. Specifically, a Z-twist was generated for the inner-wrapped nylon filaments by the first clockwise rotating hollow spindle, while an S-twist was generated for the outer-wrapped nylon filaments by the second anticlockwise rotating hollow spindle. Finally, the double-wrapped yarns were formed after passing through the drafting rollers and wound by the take-up rollers (Figure 1A,B). It should be noted that the drafting ratio was controlled by the speed ratio of drafting rollers and feeding rollers, while the take-up ratio was controlled by the speed ratio of take-up rollers and drafting rollers. To obtain double-wrapped yarns with excellent mechanical properties, we systematically explored the influence of inner wrapping density, outer wrapping density, take-up ratio, and drafting ratio on the structure and mechanical properties of the double-wrapped yarns, we also further investigated their performance as weft-lining yarns for generating compression fabrics using computerized flat-knitting machines (Figure 1C,D).

### 3.2. Effect of the Inner and Outer Wrapping Density on the Mechanical Properties of Double-Wrapped Yarns

**Figure 2:**
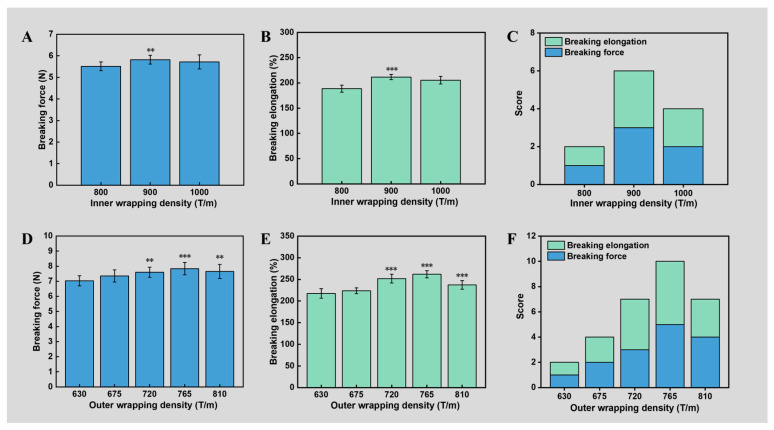
Effect of the inner and outer wrapping density on the mechanical properties of double-wrapped yarns. (**A**–**C**) Effect of inner wrapping density: (**A**) breaking force, (**B**) breaking elongation, and (**C**) queueing scoring. (**D**–**F**) Effect of outer wrapping density: (**D**) breaking force, (**E**) breaking elongation, and (**F**) queueing scoring. Symbols on the bar graphs represent comparisons with the first bar (**: sig < 0.01; ***: sig < 0.001).

Initially, we investigated the effect of the inner wrapping density on the mechanical properties of the double-wrapped yarns. Specifically, the inner wrapping density was varied from 800 T/m to 1000 T/m, while the outer wrapping density, take-up ratio, and drafting ratio were fixed at 675 T/m, 0.5, and 3.2, respectively. As the mechanical properties of yarns determine the ability of garments to resist damage from external forces, the breaking force and elongation of the yarns were quantified based on stress–strain curves. Notably, the inner wrapping density had a significant effect on the breaking force (F-value = 3.479, *p*-value = 0.047). There was no significant difference in yarn force between 900 T/m and 1000 T/m, but both were higher than 800 T/m (Figure 2A). We believe that the tensile strength is simultaneously determined by the tensile strength of the Spandex core, the friction along the axis of double-wrapped yarns, and the component force of wrapped filaments along the double-wrapped yarn axis [38]. When the inner wrapping density was increased, the wrapping angle was elevated, resulting in less component force of wrapped filaments along the double-wrapped yarn axis. In contrast, higher inner wrapping density also led to large pressure between the Spandex core and wrapped filaments, generating larger frictions along the axis of the double-wrapped yarns due to the increased number of inner wrapping filaments per length of yarn [19]. Therefore, the maximum yarn force obtained at 900 T/m inner wrapping density can be attributed to the synergetic effect of friction and component force of wrapped filaments along the double-wrapped yarn axis.

Notably, the inner wrapping density also had a significant effect on the elongation (F-value = 29.249, *p*-value < 0.001). Specifically, the elongation was 211.57% ± 5.16% and 205.40% ± 7.56% when the inner wrapping density was 900 T/m and 1000 T/m, respectively, both higher than 800 T/m (Figure 2B). The larger elongation under higher inner wrapping density can be attributed to the enhanced wrapping angle, making wrapped filaments easier to deform along the axis of the double-wrapped yarns [39]. However, too high a friction between the Spandex core and wrapped filaments generated under high wrapping density may also prevent the double-wrapped yarns from deformation. To obtain optimized conditions, we further chose the queueing scoring rule to evaluate the overall performance. Specifically, high tensile force and larger elongation were targeted for each parameter combination score. As a result, we found that 900 T/m was the best processing parameter for achieving the highest score of 6 points (Figure 2C).

In the exploration of the outer-wrapped filaments, the outer wrapping density ranged from 630 T/m to 810 T/m, while the inner wrapping density, take-up ratio, and drafting ratio were fixed at 900 T/m, 0.7, and 4.8, respectively. Similarly, the outer wrapping density also had a significant effect on the breaking force (F-value = 5.516, *p*-value = 0.001). Specifically, the highest breaking force (7.84 ± 0.41 N) was obtained when the outer wrapping density was 765 T/m (Figure 2D). Also, the outer wrapping density had a significant effect on the elongation (F-value = 3.515, *p*-value = 0.015). Maximum elongation of 261.81% ± 8.26% was obtained when the outer wrapping density was 765 T/m (Figure 2E). Notably, the elongation of yarns obtained at 765 T/m was more than 20% higher than that of 675 T/m. In addition, the queueing scoring rule showed that 765 T/m at 10 points was the best combination of process parameters (Figure 2F). It should be noted that the highest breaking force and elongation obtained at 765 T/m outer wrapping density could be explained by the same principles as inner wrapping density.

### 3.3. Effect of the Take-Up and Drafting Ratios on Mechanical Properties of Double-Wrapped Yarns

**Figure 3:**
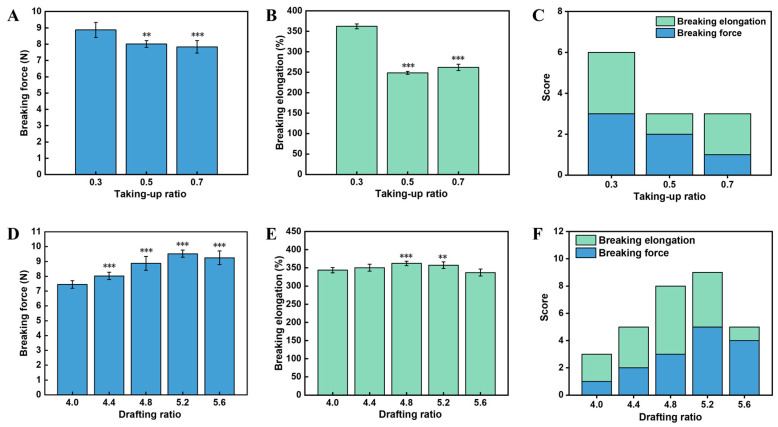
Effect of the take-up and drafting ratios on mechanical properties of double-wrapped yarns. (**A**–**C**) Effect of take-up ratio: (**A**) breaking force, (**B**) breaking elongation, and (**C**) queueing scoring. (**D**–**F**) Effect of drafting ratio: (**D**) breaking force, (**E**) breaking elongation, and (**F**) queueing scoring. Symbols on the bar graphs represent comparisons with the first bar (**: sig < 0.01; ***: sig < 0.001).

The take-up ratio affects the storage tension of the yarn, which is usually wound with slack. We therefore explored the effect of take-up ratios ranging from 0.3 to 0.7 on the mechanical properties of the yarns, while maintaining the drafting ratio and inner and outer wrapping density constant at 4.8, 900 T/m, and 765 T/m. After statistically evaluating the mechanical properties, we found that the take-up ratio had a significant effect on the breaking force (F-value = 14.502, *p*-value < 0.001) and elongation (F-value = 611.143, *p*-value < 0.001). Specifically, the breaking force was the highest (8.87 ± 0.46 N) when the take-up ratio was 0.3 (Figure 3A). In addition, the elongation of yarns obtained at the take-up ratio of 0.3 was 45.8% and 38.3% higher than that of 0.5 (*p* < 0.001) and 0.7 (*p* < 0.001) (Figure 3B). The queueing scoring rule indicated that 0.3 was the best combination of process parameters (Figure 3C). We believe that a larger take-up ratio resulted in non-recoverable plastic deformation of the Spandex core after storage in the slack, which would weaken the mechanical properties of the Spandex core [40]. Due to plastic deformation, the double-wrapped yarns would become less extensible, thus resulting in less elongation.

We next investigated the influence of the drafting ratio on the mechanical properties of double-wrapped yarns. Specifically, the drafting ratio ranged from 4.0 to 5.6, while inner and outer wrapping density and take-up ratio were kept constant at 900 T/m, 765 T/m, and 0.3. Results indicated that the drafting ratio also had a significant effect on the breaking force (F-value = 38.405, *p*-value < 0.001) and elongation (F-value = 12.827, *p*-value < 0.001). The highest breaking force up to 9.52 ± 0.24 N was obtained when the drafting ratio was 5.2, while the elongation was 357.28% ± 9.10% (Figure 3D). Also, the highest elongation was 362.14% ± 5.93% when the drafting ratio was 4.8 (Figure 3E), while the breaking force was 8.87 ± 0.46 N. The queueing scoring rule showed that 5.2 was the best combination of process parameters (Figure 3F). Notably, a higher drafting ratio would lead to finer Spandex, allowing for more fibers to be incorporated into the yarn cross section [41]. It also leads to a larger retraction of double-wrapped yarns, indirectly elevating the wrapping density for both inner and outer wrapping filaments, thereby generating yarns with better mechanical properties at a relatively higher drafting ratio. The decreased tensile strength and elongation at the highest drafting ratio of 5.6 can also be ascribed to the synergetic effect of frictions and component force of wrapped filaments along the double-wrapped yarn axis.

### 3.4. Comparison of Yarn Mechanical Properties and Knitted Fabrics

**Figure 4:**
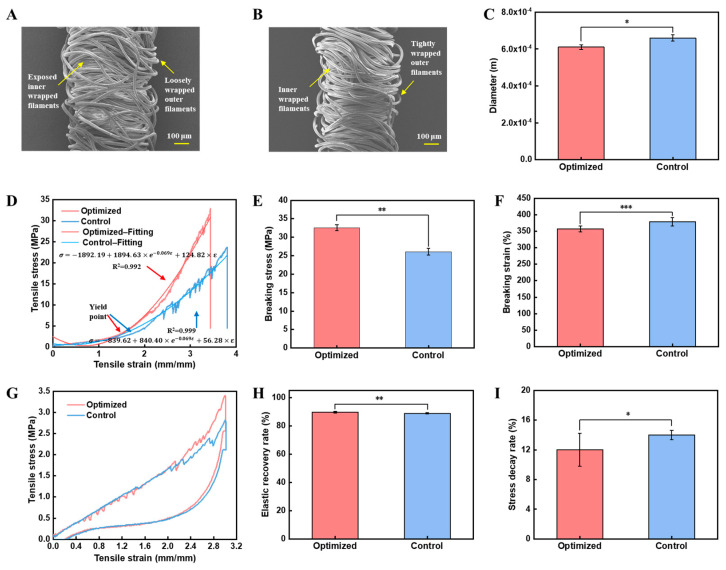
Comparison of mechanical properties of the optimized yarn with a commercially available wrapped yarn. (**A**,**B**) SEM images: (**A**) the commercially available yarn and (**B**) the optimized yarn. (**C**) Comparison of yarn diameter; (**D**) stress–strain curves; (**E**) breaking stress; (**F**) breaking strain; (**G**) elastic recovery curves; (**H**) elastic recovery rate; (**I**) stress decay rate (*: sig < 0.05; **: sig < 0.01; ***: sig < 0.001).

To further evaluate the performance of the optimized double-wrapped yarn obtained at inner and outer wrapping density, take-up ratio, and drafting ratio of 900 T/m, 765 T/m, 0.3, and 5.2, respectively, a commercially available wrapped yarn with a similar diameter was employed as a control for comparison (Figure 4A–C). Notably, the wrapping density of the control yarn was relatively low, and as a result, we can see part of the inner wrapped filaments (Figure 4A). In contrast, the optimized yarn has a uniform distribution of wrapping filaments and the yarn structure is in excellent equilibrium (Figure 4B), which is particularly suitable for use as the weft-lining yarn of weft-lined knitted fabrics.

We evaluated the tensile stress, tensile strain, elastic recovery rate, and stress decay rate. Based on the stress–strain curves (Figure 4D), we found that the tensile behavior of the optimized and control yarns exhibited nonlinear elasticity. Specifically, both yarns had an obvious yield point (σ_y-optimized_ = 3.32 MPa, ε_y-optimized_ = 150%, σ_y-control_ = 3.11 MPa, ε_y-control_ = 170%). Before and after the yield point, the curves had two different slopes. Before the yield point, the yarns had a small modulus, which would be mainly due to the elongation of the Spandex and the stretching of the wrapped filaments. After the yield point, the tensile stress increased significantly due to the elongation of the wrapped filaments. In addition, there was a small fluctuation in stress–strain curves, but the overall strength tended to increase [42,43,44]. After the yarn reaches its peak stress, the stress suddenly decreases to a small value and there is no post-peak region at these relatively high strain rates, which is consistent with theories of failure of brittle materials [45,46]. In addition, brittle fractures usually occur in Spandex and ductile fractures in nylon [47]. Therefore, we believe that the yarn changed from ductile to brittle failure during stretching [45,46].

Next, we further quantified the yarns’ mechanical properties. Specifically, the optimized yarns exhibited higher breaking stress (32.59 ± 0.82 MPa, *p* < 0.001) and a slightly smaller breaking strain (357.28% ± 8.58%, *p* < 0.001) (Figure 4D–F). Notably, the breaking strain of the optimized yarns was much higher than that of the core-spun yarns (<30%), while the breaking stress of the optimized yarns is similar to that of the core-spun yarns [20,21,22,23,24]. Importantly, our optimized yarns have higher breaking stress than other double-wrapped yarns reported [26] due to their unique design of an untwisted core of Spandex and double-wrapped nylon filaments in opposite twists. Specifically, the higher tensile stress of the optimized yarn can be attributed to the more homogeneous and dense structure of the wrapping sheath. In addition, the tightly wrapped filaments can provide more friction between the wrapped filaments and the Spandex core, while the superior breaking strain can be attributed to the untwisted core with a distinct core-to-sheath relationship.

To better analyze the yarns’ mechanical properties, we further conducted analytical calculations based on the mathematical model of the tensile behavior of our double-wrapped yarns [48]. Regarding the unique structure of our double-wrapped yarns, we believe that the mechanical properties consist of three elements (i.e., Hook’s spring, Newton’s dashpot, and Maxwell) connected in parallel. Specifically, the untwisted Spandex core can be equated to Hook’s spring because of its low energy dissipation, while the wrapped filaments have fiber sliding and can be equivalent to Newton’s dashpot. The core and wrapped filaments can slide relative to each other during stretching, and only part of the deformation can recover. Therefore, the interaction between the Spandex core and wrapped filaments can be regarded as a linear spring and Newton dashpot in series, namely, the Maxwell model. In summary, the mathematical model of the tensile behavior of our double-wrapped yarns consists of Hook’s spring, Newton’s dashpot, and Maxwell model in parallel [44,48,49]. Referring to the literature [48], we fitted the two curves based on the mathematical model for stretching of wrapped yarns as $\sigma =k\left(\eta_{1}+\eta_{2}\right)\left(1-e^{-\frac{E_{1}}{\eta_{1}}\cdot \frac{\varepsilon }{k}}\right)+E_{2}\varepsilon$ (Figure 4D). The fitting equation for the optimized yarn was $\sigma =-1892.19+1894.63\times e^{-0.069\varepsilon }+124.82\times \varepsilon$, R^2^ = 0.992; and the fitting equation for the control yarn was $\sigma =-839.62+840.40\times e^{-0.069\varepsilon }+56.28\times \varepsilon$, R^2^ = 0.999. Notably, the R^2^ value of the two fitted curves was higher than 99%, indicating that the analytical calculations and experimental data are in good agreement.

Furthermore, the optimized yarns exhibited a higher elastic recovery rate (*p* < 0.01) and a much lower stress decay rate (*p* < 0.05) (Figure 4G–I). Specifically, the elastic recovery rate and stress decay rate of the optimized yarns were 89.62% ± 0.50% and 12.0% ± 2.2%, while the elastic recovery rate and stress decay rate of the control yarn were 88.79% ± 0.48% and 14.0% ± 0.6%. Importantly, the elastic properties of our optimized yarns are superior to core-spun yarns. Specifically, the elastic recovery rate of core-spun yarns is only 55% [23] and the stress decay rate is about 15% [50]. We believe that the lower stress decay rate originated from the stabilized outer-wrapped filaments. When the outer filaments are loosely wrapped for the control yarn, the stability of the shell structure during stretching will be greatly reduced, resulting in a lower elastic recovery rate [50]. Therefore, the optimized yarn has a more stable structure, which can be used for generating compression fabrics to generate consecutive force. Nevertheless, neither yarn could fully recover to its initial length after 300% stretch, as the plastic deformation of the yarn occurs when ε > ε_y_ (Figure 4G) [30].

To verify the performance of compression fabrics fabricated with the optimized yarn, both optimized and control yarns were used as the weft-lining yarns for generating weft-lined fabrics using computerized flat-knitting machines. We envision that the optimized wrapped yarns could be used as main yarns to form looped structures if the diameter could be further decreased. In addition, the optimized wrapped yarns could also be used as both weft-lining yarns and warp-lining yarns if a warp-knitting machine is employed. In this way, we can potentially be able to generate bidirectional stretch fabrics with high elongation and strength in both directions.

**Figure 5:**
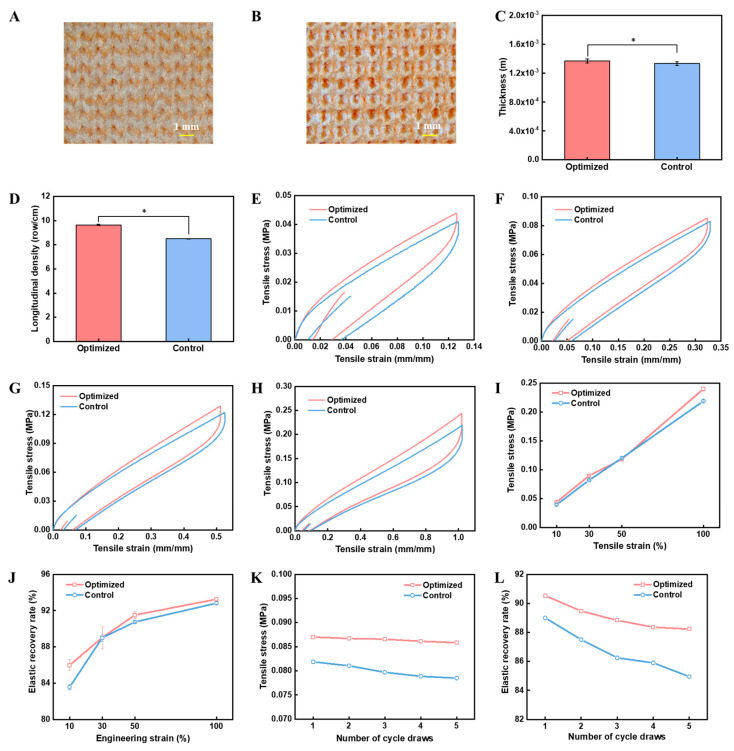
Comparison of the performance of knitted fabrics. (**A**,**B**) Images of the fabric generated using the optimized double-wrapped yarn: (**A**) front side, (**B**) back side. (**C**) Thickness of knitted fabrics; (**D**) longitudinal density of knitted fabrics; (**E**–**H**) elastic recovery curves of knitted fabrics at different strains: (**E**) 10%, (**F**) 30%, (**G**) 50%, and (**H**) 100%. (**I**,**J**) Quantification of knitted fabrics at different strains: (**I**) force, (**J**) elastic recovery rate. (**K**,**L**) Quantification of knitted fabrics for 5 cycles of stretching: (**K**) force, (**L**) elastic recovery rate (*: sig < 0.05).

Notably, both fabrics have good flatness with similar thicknesses (Figure 5A–C). In addition, the weft-lining yarns can be completely hidden. Notably, the optimized yarn resulted in a knitted fabric with a higher longitudinal density than those made from the control yarn (Figure 5D). Specifically, knitted fabrics made from optimized yarns had an average of 9.64 ± 0.06 transverse rows per centimeter, while those fabrics made from control yarns had an average of 8.49 ± 0.02 transverse rows per centimeter. It should be noted that the weft-lining yarns were under the same tension during the knitting process; therefore, the optimized weft-lining yarn would have less deformation based ono the stress–strain cure and lead to less shrinkage in the weft direction (Figure 4D) due to higher longitudinal density.

In addition, the tensile stress and elastic recovery rate of knitted fabrics along the weft direction were compared. Specifically, different strains ranging from 10% to 100% were investigated (Figure 5E–H). In general, the knitted fabrics made from optimized yarns maintained higher tensile stress and elastic recovery rates at different strains investigated (Figure 5I,J). Specifically, the tensile stress of the knitted fabrics made from optimized yarns was 0.04 ± 0.0008 MPa, 0.09 ± 0.0005 MPa, 0.12 ± 0.002 MPa, and 0.24 ± 0.004 MPa at tensile strains of 10%, 30%, 50%, and 100%, respectively (Figure 5I), which can meet the performance requirements of pressure fabric [27]. In addition, the elastic recovery rate of the knitted fabrics made from optimized yarns was 85.98% ± 0.62%, 89.04% ±1.27%, 91.51% ± 0.39%, and 93.27% ± 0.18% at tensile strains of 10%, 30%, 50%, and 100%, respectively (Figure 5J), which is much higher than that of elastic woven fabrics (70–80%) [51]. In comparison, the elastic recovery of the optimized fabrics was 91.51% ± 0.39% at 50% stretch, whereas the elastic recovery rate of the knitted fabrics made from core-spun yarns was only 60.06% at a strain of 50%, much lower than our fabrics [30]. To simulate practical conditions, we explored the changes in mechanical properties of the fabrics under 30% cyclic stretching (Figure 5K,L). After five cycles of stretching, the force and elastic recovery rate of the control fabric decreased by 4.1% and 4.5%, respectively, while the optimized fabric decreased by only 1.3% and 2.6%. The elastic recovery of the fabrics prepared from the optimized samples after five stretches was significantly higher (88.23%) than that of knitted fabrics with 43% Spandex content (80%) [52]. The optimized double-wrapped yarn could be used to fabricate weft-lined fabrics with improved anti-fatigue properties.

## 4. Conclusions

In this paper, anti-fatigue double-wrapped yarns with an untwisted core of Spandex and double-wrapped nylon filaments in opposite twists were developed to generate compression fabrics with excellent anti-fatigue performance. After systematic optimization of processing parameters of inner and outer wrapping density, take-up ratio, and drafting ratio, we were able to obtain double-wrapped yarns and knitted fabrics with excellent mechanical properties compared to recently reported data from both core-spun and wrapped yarns. We believe that our study could provide valuable guidance for the development of compression garments with excellent anti-fatigue properties. Specific conclusions are as follows.

(1) Yarns with excellent tensile stress (32.59 ± 0.82 MPa) and tensile strain (357.28% ± 9.10%) were obtained when inner and outer wrapping density, the take-up ratio, and drafting ratio of 900 T/m, 765 T/m, 0.3, and 5.2, respectively.
(2) Compared to commercial wrapped yarns and reported data about core-spun and wrapped yarns, the optimized yarns exhibited a higher elastic recovery rate (89.6% ± 0.5%) and a much lower stress decay rate (12.0% ± 2.2%).
(3) Notably, the elastic recovery rate of weft-lined fabrics based on optimized wrapped yarns can be maintained above 85.98% ± 0.62% at any elongation. The stress decay rate can be kept below 10.67% ± 0.15% after five cycles of stretching, indicating that the knitted fabrics are ideal candidates to meet the performance requirements of compression stockings and sleeves.

## Figures and Tables

**Table 1:** Characteristics of Spandex and nylon filament.

Materials	Linear Density	Force (N)	Elongation	Elastic Recovery Rate	Stress Decay Rate	Role
Spandex filament	560 D	0.84 ± 0.03 (300%)	——	87.5% ± 3.2%	10.0% ± 1.3%	Core
Nylon filament	70 D/24 f	3.87 ± 0.09 (Break)	34.46% ± 2.30%	21.8% ± 10.2%	15.7% ± 1.4%	Sheath

## Data Availability

The original contributions presented in the study are included in the article material. Further inquiries can be directed to the corresponding authors.

## References

[1] Mikucioniene D., Halavska L., Melnyk L., Milašius R., Laureckiene G., Arabuli S. (2024). Classification, Structure and Construction of Functional Orthopaedic Compression Knits for Medical Application: A Review. Appl. Sci..

[2] Ghai S., Nilson F., Gustavsson J., Ghai I. (2024). Influence of compression garments on proprioception: A systematic review and meta-analysis. Ann. N. Y. Acad. Sci..

[3] Luo Y., Liu X., Chen F., Zhang H., Xiao X. (2023). Numerical Simulation on Crack–Inclusion Interaction for Rib-to-Deck Welded Joints in Orthotropic Steel Deck. Metals.

[4] Chen F., Zhang H., Li Z., Luo Y., Xiao X., Liu Y. (2023). Residual stresses effects on fatigue crack growth behavior of rib-to-deck double-sided welded joints in orthotropic steel decks. Adv. Struct. Eng..

[5] Weakley J., Broatch J., O’Riordan S., Morrison M., Maniar N., Halson S.L. (2022). Putting the Squeeze on Compression Garments: Current Evidence and Recommendations for Future Research: A Systematic Scoping Review. Sports Med..

[6] Ye C., Liu R., Ying M.T.C., Liang F., Shi Y. (2023). Characterizing the biomechanical transmission effects of elastic compression stockings on lower limb tissues by using 3D finite element modelling. Mater. Des..

[7] Carman T.L., Al-Omari A. (2019). Evaluation and Management of Chronic Venous Disease Using the Foundation of CEAP. Curr. Cardiol. Rep..

[8] Kankariya N. (2022). Material, structure, and design of textile-based compression devices for managing chronic edema. J. Ind. Text..

[9] De Decker I., Beeckman A., Hoeksema H., De Mey K., Verbelen J., De Coninck P., Blondeel P., Speeckaert M.M., Monstrey S., Claes K.E.Y. (2023). Pressure therapy for scars: Myth or reality? A systematic review. Burns.

[10] Powell H.M., Nedelec B. (2021). Mechanomodulation of Burn Scarring Via Pressure Therapy. Adv. Wound Care.

[11] Play M.-C., Trama R., Millet G.Y., Hautier C., Giandolini M., Rossi J. (2022). Soft Tissue Vibrations in Running: A Narrative Review. Sports Med. Open.

[12] Lee H., Kim R.-K., Chae W.-S., Kang N. (2023). Compression Sportswear Improves Speed, Endurance, and Functional Motor Performances: A Meta-Analysis. Appl. Sci..

[13] Yang C., Yang Y., Xu Y., Zhang Z., Lake M., Fu W. (2024). Whole leg compression garments influence lower limb kinematics and associated muscle synergies during running. Front. Bioeng. Biotechnol..

[14] Xiong Y., Tao X. (2018). Compression Garments for Medical Therapy and Sports. Polymers.

[15] Hu J., Lu J., Zhu Y. (2008). New Developments in Elastic Fibers. Polym. Rev..

[16] Wu J., Jin Z., Jin J., Yan Y., Tao J. (2019). Study on the tensile modulus of seamless fabric and tight compression finite element modeling. Text. Res. J..

[17] Bhat G., Chand S., Yakopson S. (2001). Thermal properties of elastic fibers. Thermochim. Acta.

[18] Bansal P., Maity S., Sinha S.K. (2020). Elastic Recovery and Performance of Denim Fabric Prepared by Cotton/Lycra Core Spun Yarns. J. Nat. Fibers.

[19] Lin J.-H., Chang C.-W., Lou C.-W., Hsing W.-H. (2004). Mechanical Properties of Highly Elastic Complex Yarns with Spandex Made by a Novel Rotor Twister. Text. Res. J..

[20] Helali H., Babay A.D., Msahli S. (2012). Effect of elastane draft on the rheological modelling of elastane core spun yarn. J. Text. Inst..

[21] Waseem Ullah Khan R.M., Nawab Y., Mubeen Safdar M., Ayub Asghar M., Umair M. (2023). Comparative Geometrical Analysis of In Situ Mechanical Performance of 2-D Woven In-Plane Auxetic Structures. J. Test. Eval..

[22] Babaarslan O., Sarıoğlu E., Ertek Avcı M. (2020). A comparative study on performance characteristics of multicomponent core-spun yarns containing cotton/PET/elastane. J. Text. Inst..

[23] Babaarslan O., Shahid M.A., Doğan F.B. (2023). Design of Hybrid Yarn with the Combination of Fiber and Filaments and Its Effect on the Denim Fabric Performance. Fibres Text. East. Eur..

[24] Elrys S.M.E., Faheem El-Habiby F., Abd Elkhalek R., Eldeeb A.S., El-Hossiny A.M. (2021). Investigation into the effects of yarn structure and yarn count on different types of core-spun yarns. Text. Res. J..

[25] Lou C.W., Hu J.-J., Lu P.C., Lin J.-H. (2015). Effect of twist coefficient and thermal treatment temperature on elasticity and tensile strength of wrapped yarns. Text. Res. J..

[26] Jin Z.M., Ou Y., Xu N., Yan Y.X. (2013). Pressure Analysis on Medical Compression Hosiery and Property Research of Lycra/Nylon Double Wrapped Yarn. Adv. Mater. Res..

[27] Bera M., Chattopadhyay R., Gupta D. (2015). Influence of linear density of elastic inlay yarn on pressure generation on human body. J. Ind. Text..

[28] Coghlan N., Copley J., Aplin T., Strong J. (2019). How to improve compression garment wear after burns: Patient and therapist perspectives. Burns.

[29] Maleki H., Aghajani M., Sadeghi A.H., Jeddi A.A.A. (2011). On the Pressure Behavior of Tubular Weft Knitted Fabrics Constructed from Textured Polyester Yarns. J. Eng. Fibers Fabr..

[30] Senthilkumar M., Anbumani N. (2010). Dynamics of Elastic Knitted Fabrics for Sports Wear. J. Ind. Text..

[31] Halfaoui R., Chemani B. (2013). The influence of cyclic deformation on the strength and elongation at break of carded and combed wool yarns. J. Text. Inst..

[32] (2005). Textiles—Standard Atmospheres for Conditioning and Testing.

[33] (2013). Testing Method for Tencity of Spandex Filament Yarns.

[34] (2013). Textiles—Yarns from Packages—Determination of Single-End Breaking Force and Elongation at Break Using Constant Rate of Extension(CRE) Tester.

[35] (2012). Testing Method for Elasticty of Spandex Filament Yarns.

[36] (2004). Stretch and Recovery Testing Method for Knits.

[37] Knoth S., Amin R. (2003). Autocorrelation and tolerance limits. J. Stat. Comput. Simul..

[38] Bruniaux P., Crepin D., Lun B. (2012). Modeling the mechanics of a medical compression stocking through its components behavior: Part 1—modeling at the yarn scale. Text. Res. J..

[39] Lin J.-H., He C.-H., Huang Y.-T., Lou C.-W. (2017). Functional Elastic Knits Made of Bamboo Charcoal and Quick-Dry Yarns: Manufacturing Techniques and Property Evaluations. Appl. Sci..

[40] Falkner A.H. (1994). Bar Design for Length Compensation in Textile Winding. Text. Res. J..

[41] Hossain M.A., Hossain M.A., Emon J.H., Islam M.T. (2024). Effect of core material draft ratio and denier on core spun yarn and denim fabric properties pre and post washing. Heliyon.

[42] Farajzadeh Z., Ghasemi F., Mousazadegan F., Ezazshahabi N. (2024). Investigation of the Spike Penetration Resistance of the Weft-Knitted Fabrics with Different Elasticity Ratio. Fibers Polym..

[43] Chen J., Li Y., Yan T., Liu X., Cao J., Du Z. (2021). Influence of re-entrant hexagonal structure and helical auxetic yarn on the tensile and auxetic behavior of parametric fabrics. Text. Res. J..

[44] Yang R., Hu A., Zhang X., Liu S., Gao J., Lv Z., Zhang H. (2023). Viscoelastic tensile model of core/wrapped composite yarn with double filament. Text. Res. J..

[45] Wang H., Hazell P.J., Shankar K., Morozov E.V., Jovanoski Z., Brown A.D., Li Z., Escobedo-Diaz J.P. (2019). Tensile properties of ultra-high-molecular-weight polyethylene single yarns at different strain rates. J. Compos. Mater..

[46] Allaer K., De Baere I., Lava P., Van Paepegem W., Degrieck J. (2014). On the in-plane mechanical properties of stainless steel fibre reinforced ductile composites. Compos. Sci. Technol..

[47] Sohanaki P., Ahamadloo E., Gharehaghaji A.A., Malek R.M.A. (2022). Fabrication and characterization of three-layer nanofibrous yarn (PA6/PU/PA6). Polym. Bull..

[48] Liu S., Gao Y., Chen X., Du Z. (2019). A Theoretical Study on the Effect of Structural Parameter on Tensile Properties of Helical Auxetic Yarns. Fibers Polym..

[49] Peng L., Zheng Q., Li Y., Hu X. (2022). Application of viscoelastic-plastic model in blended yarn. Text. Res. J..

[50] Elrys S.M.M.E., El- Habiby F.F., Eldeeb A.S., El-Hossiny A.M., Abd Elkhalek R. (2022). Influence of core yarn structure and yarn count on yarn elastic properties. Text. Res. J..

[51] Jiang L., Zulifqar A., Hai A.M., Anwar F., Hu H., Liu F., Chen H. (2023). Effect of using alternate elastic and non-elastic yarns in warp on shrinkage and stretch behavior of bi-stretch woven fabrics. J. Eng. Fibers Fabr..

[52] Jovanović T., Penava Ž., Vrljičak Z. (2022). Impact of the Elastane Percentage on the Elastic Properties of Knitted Fabrics under Cyclic Loading. Materials.

